# Adopting and maintaining a healthy lifestyle in low SES families: How the experience of motherhood shapes responses to dietary and physical activity public health guidance

**DOI:** 10.1186/s12889-022-13502-4

**Published:** 2022-06-01

**Authors:** P. Wittels, T. Kay, L. Mansfield

**Affiliations:** 1 Brunel University London, Uxbridge, UK; 2 Independent Researcher, Loughborough, UK

**Keywords:** Mothers, Lifestyle behaviours, Behaviour change, Diet, Physical activity, Ethic of care, Qualitative, Low socioeconomic status, Guidance, Interventions

## Abstract

**Background:**

Public health guidance and associated interventions seek to bring about change in diet and physical activity behaviours to improve life expectancy and healthy life expectancy in the population. Low socioeconomic status (SES) groups suffer from reduced life/healthy life expectancy compared with the population as a whole. This in-depth qualitative study, investigates the lives and experiences of mothers with young children living in a low SES area in a London (UK) borough, to understand the context in which current public health guidance on diet and physical activity is received and viewed, and how this understanding could be used to inform the development of public health guidance and interventions for this group.

**Methods:**

The mothers (*n* = 20), were recruited from a Surestart Centre, Children’s Centres and through the local branch of a national charity. The mothers took part in a series of three in depth interviews over two years (2016–2017). Thematic analysis was used to generate an iterative set of codes informing an understanding of motherhood, diet and physical activity.

**Results:**

Motherhood was found to be a major factor for understanding and interpreting how women in low SES areas respond to public health guidance on diet and physical activity. The mothers were well informed about dietary guidance, considered that provision of healthy food was critical to the mothering role, but found it difficult to implement the guidance in their own lives. In contrast, physical activity was not seen as integral to the mothering role, although it was acknowledged that it played a part in ensuring that the mothers remained healthy enough to fulfil all their duties. Constrained by the ethic of care, and a lack of material and time resources, the mothers prioritised their family’s health above their own. This study, enabled the mothers to articulate ideas for interventions that would be supportive of a healthy lifestyle and of practical application in their busy lives.

**Conclusions:**

Mothers from this low SES area responded differently to the public health guidance on diet and on physical activity. Whilst providing and modelling a healthy diet was seen as an essential part of the mother’s role, participating in leisure based physical activity was problematic, and unless carefully framed, could be perceived as selfish behaviour, inappropriate for the “good” mother.

## Background

In the UK, life expectancy is related to socioeconomic status (SES) with individuals of high SES enjoying a longer life than those of low SES [1] as well as a healthier life [2–5]. The explanation for this difference in life expectancy, that has persisted despite the provision of free health care at the point of delivery through the National Health Service (NHS), lies in the social determinants of health. Health is strongly influenced by the environment and community where people live, and their material resources [6, 7]. Whilst this is acknowledged and understood, much of the public health activity designed to address health inequalities focuses not on the social determinants of health, but instead on lifestyle behaviours [7]. Lifestyle behaviours are related to the social determinants of health; they can be considered a consequence of disadvantage and therefore a product of the social determinants of health, rather than a standalone explanation for poor health in disadvantaged groups [8]. This means that to understand lifestyle behaviours, with a view to influencing them, they need to be studied in the context of the community where people live, and their access to resources.

A further problem encountered in narrowly relating public health guidance to behaviour in a low SES group is that most public health interventions rely on behaviour change theory and target the individual, despite the broad evidence base indicating that diet and physical activity behaviours are influenced by many factors beyond the individual, including peer group, local environment and policy [9, 10]. Moreover, behaviour change theories may not offer the best foundation for assessing and influencing lifestyle behaviours because of the high degree of variability in individual behaviours and the social contexts in which they take place [11, 12]. The theory of planned behaviour which is regularly used in the development of public health interventions, states that individual behaviour is driven by behaviour intentions which in turn are dependent on three factors, personal attitudes, an individual’s perception of the attitudes of others (family and friends) and the individual’s behavioural control, which is a balance of internal and external factors [13]. This theory, however is “most predictive amongst the young, fit and affluent and when predicting self-reported behaviour over a short term” ([14], p3) and may therefore be of limited value in predicting long term behaviour change in mothers from disadvantaged areas, particularly when individuals are not suffering from ill health as a result of their behaviours, and when upstream factors are preventing behaviour change [15].

In an effort to improve the diet and physical activity of the population and thereby increase life expectancy and healthy life expectancy, guidelines have been issued on diet, by Public Health England, which cover total calories to be consumed and recommended intake of macro and micro nutrients [16], and on physical activity by the Department of Health [17], which set out the time to be spent on moderate and/or intense activity, and strength training. The drive to achieve adoption of these guidelines is supported through public health promotional activities designed to reach the whole population, and specific interventions mostly aimed at the individual, and more rarely to make the environment supportive of behavioural change [8, 9]. These types of intervention and public health guidance that target the whole population are disproportionately taken up by high SES groups, thus paradoxically, further widening health inequalities [18]. Interventions targeting the individual are most likely to widen inequalities with eight of the eighteen interventions in McGill et al.’s 2015 review of interventions to promote healthy eating, including all four dietary counselling interventions, having a greater impact on higher SES groups [18].

Mothers of young children are an important target group in the population for public heath guidance and interventions for the adoption of healthy lifestyles, first because they are a large group with time ahead of them to benefit from any lifestyle changes, secondly because of their influence on the next generation through the food they provide to their families and modelling lifestyle behaviours, and thirdly for their own well-being. According to the ethic of care [19], women measure their worth in terms of the care they provide to others and are prepared to sacrifice their own health, putting the health of their children and partners before their own [20, 21]. Motherhood is a diverse experience [22] but for many mothers it is a time of emotional struggle where they are expected to set aside their own needs and to be always available to family members [20]. Whilst this dominant expectation of motherhood is idealised by society [20] mothers themselves can be left feeling conflicted and isolated by these demands [21]. The Covid 19 pandemic which occurred after this study was completed, is likely to have increased the caring burden on mothers of young children because the social changes arising from the pandemic have caused an increase in unpaid care work, that is disproportionality carried out by women [23]. Understanding the mother’s role within the household, especially as a carer, and the value she places on healthy lifestyle behaviours will be necessary to develop public health interventions to support mothers of young children.

This study sets out to understand how motherhood influences the perception, adoption and maintenance of two lifestyle behaviours, diet and physical activity, in a group of mothers of young children living in a low SES area of a London Borough (UK). Our approach was to appreciate and recognise the influence, in this group, of SES on lifestyle behaviours. Current public health guidance does not engage with inequalities and instead focuses on behaviour change by the individual, failing to take into account the impact of the social determinants of health [24]. We believe that without a thorough understanding of the multiple social influences on the health behaviours of mothers of young children, living in a disadvantaged area, it will not be possible to design and implement public health interventions to encourage and support the adoption of healthy lifestyle behaviours in this group.

## Methods

This interview based research used a mainly explorative interpretive methodology [25], but also drew on critical theory to acknowledge that the lives of the participants were shaped by the area in which they lived and because the study sought to collect data that could change the lives of the participants [25]. The lead researcher (PW) employed a reflexive stance throughout to acknowledge her influence on the research process and collection of data [26], built a relationship with the interviewees and explored comments made in earlier interviews, so that the data collected can be considered to be co-produced by the researcher and the participants [27].

### Recruitment of participants

Mothers were recruited through a Sure Start Centre, Children’s Centres and HomeStart, a national charity that supports families with young children that are struggling, for example feeling isolated or experiencing money problems. One of us (PW) volunteered with HomeStart and this provided a reassurance to the mothers and to the managers of the Sure Start Centre and Children’s Centres, and facilitated recruitment. The Sure Start Centre and Children’s Centres where the participants were recruited were all located in one London Borough (UK) in areas of below average SES as defined by the Index of Multiple Deprivation (IMD). The IMD is a UK measure of seven deprivation domains namely income, employment, health and disability, education and skills, housing, living environment and crime, linked to domestic post code. Twenty mothers agreed to take part in the research, two recruited from HomeStart, eight from the Sure Start Centre, nine from the Children’s Centres and one was the sister of another participant. All potential participants were provided with full information about the study explaining what participation would involve and making it clear that it was voluntary and would not impact on access to services. All participants were assured of anonymity and understood how the findings would be used. The participants gave their written informed consent in the presence of the lead researcher (PW) and were offered £20 for each interview in recognition of the time they gave to the research.

 Full Ethical approval was obtained through the Research Ethics Committee of the Department of Life Sciences at Brunel University (Reference Number RE48-14).

### Description of participants

The participant demographics are provided in Table 1 below. Culturally sensitive pseudonyms have been used in place of the actual names to protect the identity of the participants. All the participants except Yasmine had at least one child under five years old (Yasmine had an eleven-year-old child). The range of ethnicities of the participants is typical of the local population.

**Table 1:** Participant demographics

Interviewee (number of interviews)	Age	Ethnicity	No of children	Living with partner
Sumi (2)	39	Indian British	1	yes
Fatima (3)	25	North African	1	yes
Naseem (3)	40	Pakistani Danish	3	yes
Vicki (2)	32	White South African	1	yes
Radhika (2)	31	Indian	1	yes
Aahna (1)	29	Indian	1	yes
Eva (2)	31	White Eastern European	1	yes
Christine (2)	42	Filipino	2	yes
Jade (3)	37	Indian British	2	yes
Rozina (1)	32	Indian	1	yes
Shabnam (3)	44	Pakistani British	2	yes
Eisha (3)	24	North African	1	yes
Nori (3)	29	Pakistani British	3	yes
Kirti (3)	37	Indian British	3	yes
Lilly (1)	34	Sri Lankan British	1	yes
Meenakshi (3)	32	Indian British	1	yes
Rachel (3)	41	White British	1	no
Jaswinder (2)	29	Indian British	1	yes
Alison (3)	43	White British	1	yes
Yasmine (1)	43	Pakistani British	1	yes

### Data collection

Data collection was through a series of three in-depth interviews [28] and took place between January 2016 and September 2017. An interview guide was developed for each of the first, second and third interviews, with each successive interview guide building on information collected in the earlier interview. The first interview allowed a relationship to be established and focused on collecting factual information about the participants, their families and their lifestyles and introduced the topic of lifestyle behaviours. In the second interview, some of the earlier questions were repeated and some topics were discussed in more depth, particularly changes that becoming a mother had had on diet, physical activity and their health, barriers and facilitators to the adoption of healthy lifestyles, and whether they felt knowledgeable about current public health guidance. The second interview was also used to enquire about changes that had occurred since the first interview, about their priorities as a mother, being a role model and the type of interventions, they may find useful. The third interview further developed the same themes with a greater emphasis on ideas for public health interventions. After the first three first round interviews, the interview guide was amended in the light of the data collection experience. The main change was to the order of topics so that more specific questions on family meals and eating experiences preceded more general questions on health. In the third interview pictures were provided to illustrate four different ideas for public interventions that had arisen during the earlier interviews and these served as a basis to talk around the intervention concept and how it might work in practice. The ideas and the pictures were; a self-help group illustrated by a diverse group of young women talking over coffee, support for the whole family illustrated by a dual heritage family with three young children, hands on support for the family in the home illustrated by a female volunteer sitting on a sofa with a mum and baby, and changing the environment illustrated with a montage of pictures of fast food, sweets, crisps, a creche and early years education. The interviews (which were all conducted by PW, a mature PhD student and mother of three grown up children) mainly took place in the participants’ homes, an indication of the trust that was established between the participants and the researcher. PW was treated as a welcome guest and was introduced to other family members and shared food [29, 30]. Some interviews took place in a Children’s Centre, coffee shop and local leisure centre, and one was over the phone, all depending on agreeing a convenient time and place for interviewer and interviewee. In many of the interviews the participants’ small children were present and in one interview the participant’s husband was present.

An active awareness of how the relationship between the researcher and the participants can influence the research process and data collection was maintained during all the interviews [31]. The data generated came from the researcher, the participants and the interaction between the two [26]. The multiple interviews allowed the relationship between the participant and the interviewer to grow and develop leading to the disclosure of more personal information and discussion of more sensitive topics, as well as allowing the interviewer to see if responses were consistent or whether the participants’ views changed over time [32]. At the third interview the pictures were used to elicit deeper responses on how potential interventions supported the mothering role.

The interviews were conducted in 2016 and 2017 and since that time there have been changes to guidance and policy in the UK. Physical activity guidance was updated in 2019 to include specific guidance in pregnancy and after giving birth, and at the same time the guidance on strength training was reinforced. The Soft Drinks Levy was announced in March 2016 and enacted in April 2018.

Twenty participants took part in the first interview, sixteen in the second interview and ten in the third interview. Two participants moved away between the first and second interviews and were lost to follow-up, and a further two participants remained in contact but decided not to participate further. As the focus of the third interview was about potential interventions, only participants that had expressed an interest in ideas for public health interventions were approached for a third interview. The average time between the first and second interview was nine months, and between the second and third interview five months. The number of participants and the opportunity to conduct repeated interviews in a longitudinal study enabled us to mine the field in our study rather than scratch the surface and make sense of what mattered to our participants in our theoretical analysis [33]. Following the extant literature on the question of how many participants are enough for qualitative research we argue that our participants provided us in-depth data about healthy lifestyle behaviours, and our methods allowed us to revisit topics in subsequent interviews to provide further detail where required, and it is this approach that helped achieve data saturation.

The material provided to the participants made it clear that the interviews were part of a university research project and some of the participants took an active interest in the research and asked about progress at subsequent interviews.

The interviews were recorded using an Olympus DS-50 Digital Recorder. The average duration of the recorded interviews was 48 min, 52 min and 53 min for the first, second and third interviews respectively. The first interview took place in January 2016 and the last in October 2017. The transcribed interviews were not returned to the participants for comment, but points raised in earlier interviews were discussed with the participants in subsequent interviews in order to develop the depth and accuracy of our data and support our theoretical analysis.

#### Data analysis

The interviews were transcribed verbatim by PW and entered into NVivo v10 (QSR International). The approach to analysis followed that recommended by Creswell [34], starting with a thorough review of the transcript text and the preparation of an initial set of descriptive codes by PW, which were discussed and reviewed by all three authors. Through the discussion, further immersion in the data and more data collection, the coding was revised moving from the initial descriptive codes to a revised set of interpretive codes, to the identification of a principle finding and themes following the principles of reflexive thematic analysis [35] and the pathway set out by Miles and Huberman [36], see Table 2. The lead researcher was deeply immersed in the data and through discussing her ideas about shared patterns of meaning across the data set, she was able, with the help of her co-authors, to identify links that addressed the underlying research questions, but were much more profound than a shared topic and more sophisticated than the data collection questions.

 Experience and ideas from the participant interviews were fed into new participant interviews to facilitate information sharing and to allow an informal response to the findings to be collected. The participants were not asked to respond formally to the findings.

## Results

The results reported here all reflect the principle finding that motherhood influences health behaviour. We define the complex and contested nature of the motherhood-health behaviour nexus as “The Conflicted Mother”.

**Table 2:** The Conflicted Mother, themes and codes

**Coding Tree for the Conflicted Mother Principle Finding**	**-**	**-**
**Themes**	**Interpretive Codes**	**Descriptive Codes**
Prioritising Children and Family	Resources	Time
-	-	Financial
-	Prioritising Others	-
-	Multiple responsibilities	Children
-	-	Partners
-	-	Other family members
-	The guilty mother	-
-	The isolated mother	-
Challenges of putting dietary advice into practice	Diet and mothering	Undermining mothering role
-	-	External environment
-	-	Family preferences
-	-	Dietary objectives
-	Cooking at home	Cooking for children
-	Understanding of guidance	-
-	Inability to follow guidance	-
Disconnection from physical activity guidance	Guidance knowledge	Sedentary versus activity
-	-	Types of PA
-	-	Strength training
-	Relevance to mothers	-
-	Impact on family life	Family time
-	-	Enjoyment
-	-	Chore/task
-	-	Maintain health
-	Exercise for pleasure	-
Modelling healthy behaviours	Weaning period	-
-	Healthy food for all the family	Reduction of junk food
-	-	Barriers to achievement
-	-	Benefit for all family members
-	Physical activity participation	Facilitate for children
-	-	Time resources
-	-	Material resources
-	-	Access

### Principle finding: the conflicted mother

 The mothers that took part in this research were aware of healthy lifestyle advice and had a broad knowledge of dietary guidelines and the importance of a balanced diet. They were able to discuss in general terms what constituted a healthy diet. They saw it as their responsibility to provide healthy meals for their families but faced a number of challenges in doing so. As a result, their own diet often suffered. The response to physical activity guidance was more complex; they did not have enough understanding of the current guidance to make use of it to adopt healthy behaviours (particularly the nature of the recommended physical activity they should be undertaking) and questioned its applicability to their lives. Whilst diet was seen as part of the mothering role, physical activity was perceived as something separate. For some it was a further chore, a necessity to keep healthy to look after the family. The detailed results are presented under the four themes, below. The repeated nature of the interviews allowed changes in participant response over time to be noted. Overall, there was considerable consistency for each participant across the series of interviews. Even though some of the interviews were more than a year apart, the participants gave the same or similar examples to illustrate lifestyle behaviours. For some participants a healthy diet became more difficult as they went through transitions for example, when children moved from being babies to toddlers. Physical activity in the form of walking briskly, for example, became more difficult when children started walking rather than being pushed in a pram. The use of repeated interviews also allowed ideas for interventions that fitted the participants’ core identities as mothers to be explored. In the discussion they were able to talk about and challenge existing guidance and support, and offer insights that could inform further work on coproduced interventions that would have the potential to have a critical public health impact in this group.

### Prioritising children and family

Mothers described how their needs were secondary to those of their families using phrases such as “being on the back burner” (Sumi, Jade and Shabnam) to refer to their needs. The mothers understood the requirement to adopt a healthy lifestyle for themselves, but had insufficient time and material resources to meet their own needs, and those of their children and partners. When they had to choose, the mothers put their families first:

“so there are days when I don’t look after myself because I’m just too tired to do that. I make sure everybody else is fed and then put myself last” Yasmine

“It’s an awareness, it’s in the back of my head that I have to eat some foods in order to keep myself healthy but it’s not no it’s not a priority. Ever since I had him [her son] it’s not been a priority ….I think that’s purely it why I don’t eat healthily because I just want something that’s quick, satisfying and then I can forget about it and get on with the day. I think that’s why I eat unhealthy” Noorie

The study participants reported struggling to manage multiple responsibilities, that they experienced conflict trying to undertake all the tasks assigned to them, or which they assigned to themselves, and as a result, they felt guilty when they failed to live up to the high standards, they had set themselves. For example, Vicki talking about not managing time to go to an exercise class:

“every time I don’t go I feel guilty and it’s all misplaced guilty you know” Vicki.

And Christine talking about how she feels when she has a Chinese takeaway meal rather than cooking the meal herself:

“Chinese um after that I feel guilty. When I ate too much. Because because it’s not my routine eating something very oily takeaway yes I feel guilty”, Christine

They experienced guilt when they perceived themselves as taking time and resources away from the family to follow a healthy lifestyle, and when they failed to follow a healthy lifestyle, because of a lack of time and resources. The participants, for the most part, felt alone in managing their responsibilities and neither expected nor received support. Some participants did have some help from their husbands, but many did not. For example, Eisha described how when she came home from hospital after the birth of her second child earlier that day she had to “stand and cook some food for me and for my family” and Jade described how she considered giving her baby to her husband to look after so that she could exercise but decided not to because “you feel guilty…. And you don’t want that feeling”. Many of the participants also reported feelings of isolation which were compounded by their lack of resources to access activities that might help them.

### Challenges of putting dietary advice into practice

The mothers’ understanding of what the “good” mother should be achieving in terms of diet and physical activity both for themselves and as a role model for their children differed for the two life style behaviours, diet and physical activity. Following and providing a healthy diet was seen as an integral part of the mothering role.

“I don’t want him to be overweight or anything like that so I try I give him [her son, who had recently been weaned] lots of vegetables, I make it a bigger portion so we all have the same thing … I steam broccoli and cauliflower… I try to vary it so that he’s got a taste of everything” Lilly

The women were generally well informed about current public health guidance on healthy eating. They were familiar with the advice to eat five portions of fruit and vegetables a day. One or two participants were able to quote specific figures such as 6 g of salt being the recommended maximum daily allowance, but most spoke in general terms about wanting to increase their intake of fruit and vegetables and reduce fat, sugar and salt in the diet.

“they say eat lots, eat as many as you can vegetables there’s no limit to how many you can intake, the fruit, they did used to say five a day, then I’ve heard it seven a day … cut down on fat, cut down on sugar” Sumi

They used this dietary knowledge to set themselves targets of the type of food they should be eating and providing for their family, and felt guilty, when in their eyes, they fell short of this objective and failed to be good mothers. Despite this knowledge, their everyday tasks often prevented them from eating healthy meals, for example Sumi despite being very familiar with the current dietary recommendations said:

“I obviously end up eating something quick and easy, and then it might not be the healthier option”. Sumi

“it’s just you know mums are so busy um you know they’ve got school runs to do little one at home and it’s just so quick and easy just to put something in the oven and you know something very very quick and you know instead of standing there and doing something from scratch”. Shabnam

The mothers stated clearly in the course of the interviews that it is not lack of knowledge or ignorance of the public health guidance that prevents the women from eating a healthy diet. They are cognizant of the constant messaging as to what constitutes a healthy diet. They reported that because these messages are transmitted without the provision of appropriate tools to support them in adopting a healthy diet, that they are not always able to act on the messages and this engenders a feeling of guilt, and undermines their mothering role. In some cases, the environment in which they lived with easy access to a wide range of fast-food outlets and regular supermarket discounts on energy dense foods, together with family preferences for unhealthy choices, made their task even harder.

“my husband because when he wants to eat meat they [her children] also want to eat meat … when I have made um errr maybe um lentils then sometime my husband buy kebabs from outside and then they don’t want to eat lentils they want to eat kebabs” Naseem

They felt that all these difficulties are unacknowledged in the public health messages aimed at them as mothers.

### Disconnection from physical activity guidance

In contrast to the way they took note of the dietary guidance, the mothers responded very differently to the physical activity guidance. Unlike their broad knowledge of dietary guidelines, the mothers either did not know, or understand the current physical activity guidelines and they had confused the messaging on physical activity and sedentary behaviour. As women with young children, they saw themselves as active, because they equated being busy with being physically active. They considered that because they were not sedentary, they were doing enough physical activity, and that any other official advice had limited relevance for them. They therefore did not feel that a lack of time spent on physical activity had a negative impact on their mothering role. In fact, rather than feeling remorseful about their lack of participation in physical activity, most of the participants saw taking time away from their family for individual physical activity as a selfish activity, and offered that as an explanation for not exercising.

“I think it is important but if you have time or if you like something … it’s not a priority, no”. Eva

“There’s always something that comes up and I think no … No so exercise unless it’s yeh unless it’s something you have to do or enjoy doing” Jade

“I think for some people that need that yeah I think it’s important to exercise. But some women don’t need it” Noorie

The Leisure Centres in the area where the research was carried out have crèche facilities, and the participants were aware that they could leave their children in a crèche and exercise. None of the participants however chose to do this, and most of those that talked about the crèche facilities made it clear that they saw it as their responsibility to look after their children. In their eyes the good mother does not leave her child in a crèche whilst she exercises.

“I wouldn’t have left him in a crèche to go swimming even if it was available” Fatima

“I probably wouldn’t leave her there. I’m quite a…no I’d feel really bad I’d feel bad that I’m putting her in a crèche while I’m doing something leisurely if that makes any sense. I’d feel really bad” Kirti

Moreover, some of the mothers felt it would be inappropriate to spend their limited family money and use their valuable time on what they perceived to be a selfish activity.

There were two women in the study group (Aahna and Rozina) who were regular gym users, and they had both found a way to take part in physical activity, without impacting on their family responsibilities. They talked about their participation in physical activity as something they did for the family’s benefit.

“I don’t feel like going every day. But I have to go just to keep myself fit. It is really very tiring though. Going to the gym every day. It’s very tiring.” Aahna.

“So I started exercising. That’s the reason I joined. To be healthy. I have to be healthy for her [referring to her daughter].” Rozina

Aahna and Rozina both went to the gym early in the morning before their husbands and children were awake, so the sleeping husbands had responsibility for the sleeping children, and the women returned home ready to take up the care of the family before either their husband or child woke up.

### Modelling healthy behaviours

The same contrast between diet and physical activity behaviours was seen when it came to modelling healthy behaviours for their children. For many of the mothers the weaning period had been important as they wanted to introduce their children to a wide range of healthy foods particularly fruit and vegetables and many of the women had made the decision to prepare this healthy food for the whole family so that the child could see eating this food as normal.

“I do want to be a good role model to them especially when it comes to eating because I do want them to be healthy you know. I don’t want them to think it’s OK just to eat whatever you want because you have to start educating them from a young age.” Shabnam

Rozina explained how she had changed her diet because she wanted her daughter to see her eating healthy food:

“After she is born I am more conscious I want to be healthy for her. Because if I am not healthy she will ….eat whatever I eat so I try not to eat junk. That’s the reason”. Rozina.

The mothers acknowledged the difficulties in modelling a healthy diet, but thought that it was worth the effort:

“It’s not easy, it is difficult, believe me it is difficult but I make time because I know in the long run it’s healthier for my children, it’s healthier for me and my husband and I’m instilling these you know these things into my children”. Kirti

Physical activity, in contrast, was seen by the mothers as an optional activity, mainly taking place outside the home, and some of the women questioned whether it was even something they should be doing. Most, but not all the mothers, saw physical activity as outside their maternal role and therefore it was not prioritised. Some of the participants, that had very busy lives, did not think it was realistic to serve as a role model for physical activity. They acknowledged that this would be the case in an ideal world, but in their lives, they did not have the time. Christine described how she facilitates physical activity for her children rather than modelling it herself:

“They do all these things without me. Without me in the sense that my daughter she goes to swimming lessons I’m not in the pool with her. She goes to the dance classes. I take them so I think they are they get the message that it’s good to do it.” Christine

The oft repeated response by the participants of lack of time to engage in their own healthy lifestyle behaviours led to a discussion on what type of intervention the mothers would find supporting for the adoption of healthy lifestyle behaviours and realistic in the light of their busy lives.

### Listening to mothers: the potential for coproduced interventions

 The repeated and in-depth nature of the interviews provided the participants with an opportunity to reflect on and talk about both the reason why current lifestyle interventions are not serving them and the type of intervention, mode of delivery and support mechanisms that would be more helpful to health and wellbeing enhancement in their particular circumstances. All the ideas for interventions came from the participants in the first two interviews and were collated and shared by PW with the ten mothers who took part in the third interviews. The mothers’ ideas for interventions were based on their own experience of what had worked well for them and what had worked less well. The mothers took up ideas suggested by others (and presented to them during the interview) and developed them, again with reference to their personal experience and circumstances, so that the ideas for intervention were born of collaborative effort. The mothers provided insightful feedback on how the intervention ideas would fit in with their mothering role. They saw little need for more leaflets and educational materials to be presented to them, viewing such items as too general and not relevant, but perhaps more importantly they had little motivation to read this type of material. They were looking for information to be provided in a more engaging and practical way, tailored to their specific needs as mothers of young children. Their comments, with examples below, set out how the ideas for interventions matched their concept of motherhood and their core identity as mothers.

Meenakshi talked about the importance of interventions being for the whole family so that the mother feels supported rather than having to carry the burden alone: “I think they should encourage it more the whole family so at least the whole family hears the whole idea and at least they can work together to follow whatever it is…. so they can work together as a team”.

Jade spoke about the value of volunteers visiting the home to provide hands on practical support with the daily demands of providing for a family: “it would help as well having volunteers to help with ideas on what to have [to eat] because I think that’s the main problem that you have all this stuff in the fridge and you open it and you think I’ve got nothing to eat” Jade.

 Kirti voiced the importance of information coming from somebody who had shared similar experiences and ideally in a group setting “…somebody who can guide me, talk to me, answer questions motivate me you know, same might be with other mums as well or other people” Kirti.

## Discussion

The data reported here cover the principle finding that the character and experiences of motherhood influence lifestyle behaviours in a group of mothers with young children living in a low SES area in the UK. The longitudinal nature of the research and the quality of the relationships developed between the interviewer and the participants allowed critical discussion of public health interventions and the development of ideas about how public health professionals and policy makers could better address their needs. This research is therefore significant in providing a critical public health impact for the mothers who participated in the study, and more broadly for mothers living in areas of deprivation.

The relationship between SES, poor health and poor lifestyle behaviours has persisted over time and despite an improvement in health provision and the introduction of guidance and interventions aimed at reducing health inequalities, it has proved difficult to address. Whilst this in a large part is due to material and structural parameters, there are other factors at play that may be modifiable [9, 18, 37]. Thus, for example the response to the Marmot report [6] with its highlighting of psychosocial factors, led to an increase in interventions around well-being [8]. The research reported here and conducted with a group of mothers living in a low SES area, suggests that with an improved understanding of the response to lifestyle advice and in particular the impact of the mothering role, that there are other factors that can be addressed to support healthy lifestyle behaviours in low SES mothers and, in this way, reduce health inequalities.

The lifestyles behaviours of the mothers that participated in this research were shaped and, in many cases, defined by their mothering role and the context of their low SES status. This is most clearly exemplified in the lack of priority they gave to their own health needs and the lack of support they receive for prioritising themselves, despite acknowledging that they need to maintain a certain degree of health to fulfil their role as mothers. Where they do not follow lifestyle advice, it is not because they are unaware of the public health guidance, it is because the guidelines are not compatible with their mothering responsibilities and the multiple tasks they have to undertake. They are constrained by their low SES environment in both their external and domestic spheres. The mothers in this study demonstrated how the capacity of mothers in low SES areas to adopt recommended health behaviours can be impacted by undertaking a disproportionate share of housework, childcare and cooking for the household [38].

An important finding of this research is that the mothers that took part, respond to guidance on diet differently from how they respond to guidance on physical activity. The mothers have a good understanding of current dietary advice, accept its value to them as individuals and for their families, see it as an essential part of being a good mother and therefore seek to follow it in their lives. Providing and modelling a healthy diet is an integral part of being a good mother. Nevertheless, they find it difficult in practice to follow the dietary advice, particularly for themselves because of other priorities arising from their mothering role. It appears that this group of mothers, struggle to follow public health guidance. This is predicted by behaviour change theory which suggests that behaviour change is less likely to occur in low SES groups [14]. Moreover, changing behaviour for this group is difficult because of the environment in which they live, in particular the easy access to less healthy fast-food options [39], and the lack of support from family member for healthy eating [15]. Behaviour change theory addresses individual motivational factors and although in theory, it recognises the interaction of these with other influences, in practice, it does not address the specific and immediate practical impacts of the constraining factors that this study has brought to the fore. These factors together may explain why dietary intervention are disproportionately taken up by higher SES groups and therefore tend to increase inequalities [19]. The mothers behave differently with physical activity advice because undertaking exercise is not seen as an important facet of motherhood. Other studies have reported how women negotiate time away from their families to undertake physical activity [40, 41]; the unexpected finding from this research was that some of the mothers saw undertaking physical activity as a potential detraction from their mothering activities and responsibilities, and incompatible with being a good mother. The small number of mothers in the research that did undertake physical activity, justified it to themselves as being part of their mothering role, rather than something they did for themselves, and organised their physical activity so as not to detract in any way from their mothering responsibilities, for example exercising before the family wakes up, a finding also reported by Brown et al. [40] studying low SES mothers in Australia. This situation is made more complicated because the mothers are less aware of the physical activity guidelines, are confused about the messages and do not think they apply to them as mothers of young children. This knowledge of the different responses to the diet and physical activity guidance, together with an understanding of the multiplicity and complexity of the lives of low SES mothers, is a starting point for the development of appropriate interventions.

The mothers in this study are clearly following an ethic of care [19] and do not believe they have an entitlement to time or resources for themselves. The gender equality that is being accepted in the workplace does not appear to be mirrored in their homes [42], leaving the mothers with the full burden of responsibility for the home and children, and in many cases, having to choose between providing a healthy diet for their children, and eating healthily themselves. In the case of physical activity, lack of time and resources, provided only part of the explanation as to why the mothers did not exercise. Most of the participants saw taking time away from their family for individual physical activity as a selfish act, contrary to the ethic of care, and offered that as an explanation. The mothers in this study used the term “good” to describe their aspirations and self-assess their mothering practices but we did not attribute a value-judgement to the word in our analysis. Self-defined “good” mothers prioritise their families, making participation in physical activity problematic for the good mother [43, 44]. When physical activity is undertaken it may not be for pleasure, instead it becomes another task or “third shift”, which extends the duties of the ethic of care to include being fit and healthy [45].

An understanding of the barriers to the adoption of healthy lifestyle behaviours experienced by particular groups can help in the development of appropriate public health interventions. The findings reported here provide evidence of the importance of social context in seeking to understand and modify behaviour [11, 12] and also the potential limitations of the theory of planned behaviour in this demographic [14]. Our findings are supported by the qualitative work in the USA of Greenhalgh and Carney [15] who found that targeting low SES women with more information when they are isolated and stressed as a result of their multiple responsibilities in the home, is unlikely to achieve behaviour change, particularly when individuals are not currently suffering ill health as a result of their behaviour. Our findings are also in agreement with qualitative studies from other countries (Australia, Ireland, New Zealand and Turkey) which found that low SES women, constrained by the ethic of care may need support to negotiate time away from their caring responsibilities to adopt healthy lifestyle behaviours [41, 46–48]

The difference we report in participant response to public health guidance for diet and physical activity should be taken into account in the design of public health interventions for low SES mothers. In the social cognitive model of health [49], much used in health research, it is accepted that to change, an individual needs not only to understand the benefits of change and the risks of not changing behaviour, but the individual must also have the ability to change, which depends on the facilitators and barriers in the environment. Moreover, an outcome that is highly valued will have a greater influence on behaviour than a lower valued outcome. The research reported here suggests that a healthy diet is a valued outcome, and that low SES mothers have a good understanding of the benefits and risks around diet, although they have to overcome barriers in their environment to achieve a healthy diet. In contrast, physical activity is not a desired outcome for many of the low SES mothers, and where the mothers strive to undertake physical activity, it is as part of their caring role, rather than as a pleasurable leisure time pursuit.

The mothers were all, to some extent, dissatisfied with the support they currently receive to help them adopt healthy lifestyles, and during the interviews voiced many ideas and proposals for the type of support they would value to support a healthy diet and participation in physical activity. They felt that current interventions were not sufficiently tailored to their needs, but had ideas for interventions of their own, as well as being supportive of ideas from other mothers that participated in the research. Relevant and potentially effective interventions would, in their view, have to be designed to support their mothering role and accommodate the multiple demands on their time. There are examples of successful public health interventions that have been developed from lay expertise and relied on community experience, for example the Liverpool based “Fag Ends” that relied on social rather than medical support for smokers trying to stop smoking [50]; this type of intervention was found to reflect the complex lives of the recipients of the intervention [50]. In the field of physical activity, the importance of codesign of interventions with inactive people, taking into account the many barriers they have to overcome to participate in exercise, is recognised [51]. The “Well London” group is working to coproduce public health interventions for disadvantaged groups including in the areas of diet and physical activity; its approach involves a full assessment of local needs and peer to peer approaches for delivery (http://www.welllondon.org.uk/5/the-framework.html). The contribution from a critical public health perspective of the research reported here, is to show what can be achieved by developing long term trust-based relationships with the potential recipients of public health interventions. By listening to mothers and working with them to develop ideas for interventions, practical solutions tailored to their needs can be formulated. Further research is required to design and test this type of intervention.

There are some limitations to this study. The research findings reported here only apply to the specific participant group and are not representative of a demographically defined population. All the participants lived in a low SES area and had young children but they varied in age, number of children, ethnicity, income, education, health status and hours worked outside the home. The group was self-selecting, possibly agreeing to take part in the research on lifestyle behaviours because it was a topic of interest to them and they saw themselves as role models. Alternatively, they might have agreed to take part because they felt that their current behaviours were poor and hoped that they would learn something of value by participating. The mothers that took part were willing to share intimate and detailed life experiences with the interviewer (PW). The plan was to conduct a series of interviews, but this was not possible with all participants, some mothers were lost to follow-up and some were not interested in all the research stages. This is the reality of dealing with a group of people with busy lives, some of whom were only resident for a short time in the study area. In our analysis we did not consider the impact of the number and age of the children on lifestyle behaviours. A study focusing on the impact of the transitions of motherhood on lifestyle behaviours would make an interesting topic for further research. Furthermore, there is scope for a study to explore some of the more recent changes in public health guidance on physical activity, including that pertaining to pregnancy and post-partum, the acceptability and attitudes to strength training, and the impact of the Soft Drinks Levy.

## Conclusions

This qualitative in-depth study of a group of twenty mothers of young children, living in a low SES area, revealed that current public health guidance on diet is well understood, but knowledge of physical activity guidance is less good with the mothers questioning its relevance to their busy lives. The mothers experienced feelings of guilt for failing to provide a healthy diet for their families and themselves and prioritised the needs of their children above their own. Participating in physical activity was seen as a selfish activity unless it could be framed as essential to maintain health to perform the caring role. Only through understanding the experiences and feelings of low SES mothers can public health interventions be designed to support this group in adopting and maintaining healthy lifestyle behaviours. The provision of more information on diet and physical activity will not result in positive changes. Instead, the women are seeking support that takes into account the pressures of their lives and provides them with the tools to make changes. In the case of physical activity this includes demonstrating that it is compatible with being a “good” mother.

## Data Availability

The data that support the findings of this study are available in the PhD thesis Wittels PY Shaping health: understanding and influencing lifestyle behaviours in low socioeconomic women, Brunel University London, 2021 https://bura.brunel.ac.uk/bitstream/2438/21644/1/FulltextThesis.pdf .

## References

[1] Office for National Statistics. Statistical Bulletin - Inequality in healthy life expectancy at birth by national deciles of area deprivation: England, 2009-11 (2014). Retrieved from https://www.ons.gov.uk/file?uri=%2Fpeoplepopulationandcommunity%2Fhealthandsocialcare%2Fhealthandlifeexpectancies%2Fdatasets%2Finequalityinhealthylifeexpectancyatbirthbynationaldecilesofareadeprivationengland%2F200911/inequalityinhealthylifeexpectancyatbirthbynationaldecilesofareadeprivationengland200911_tcm77-356308.xls

[2] Dugravot A, Fayosse A, Dumurgier J, Bouillon K, Rayana TB, Schnitzler A, et al. Social inequalities in multimorbidity, frailty, disability, and transitions to mortality: a 24-year follow-up of the Whitehall II cohort study. Lancet Public Health. 2020;5(1):e42-50. 10.1016/S2468-2667(19)30226-9 10.1016/S2468-2667(19)30226-9 PMC7098476 31837974

[3] Head J, Chungkham HS, Hyde M, Zaninotto P, Alexanderson K, Stenholm S, et al. Socioeconomic differences in healthy and disease-free life expectancy between ages 50 and 75: a multi-cohort study. Eur. J. Public Health. 2019;29(2):267–72. 10.1093/eurpub/cky215 10.1093/eurpub/cky215 PMC6426044 30307554

[4] Public Health England. Health profile for England 2018 Chap. 5: Inequalities in health. (2018). Retrieved from https://www.gov.uk/government/publications/health-profile-for-england-2018/chapter-5-inequalities-in-health

[5] Public Health England. Health profile for England 2018 Chap. 1: Population change and trends in life expectancy. (2018). Retrieved from https://www.gov.uk/government/publications/health-profile-for-england-2018/chapter-1-population-change-and-trends-in-life-expectancy

[6] Marmot M. Strategic review of health inequalities in England Post 2010. Marmot review final report. University College London: London. (2010). Retrieved from http://www.instituteofhealthequity.org/resources-reports/fair-society-healthy-lives-the-marmot-review/fair-society-healthy-lives-full-report-pdf.pdf

[7] Marmot M, Allen J (2014). Social Determinants of Health Equity. Am. J. Public Health.

[8] Bambra C, Smith KE, Garthwaite K, Joyce KE, Hunter DJ. A labour of Sisyphus? Public policy and health inequalities research from the Black and Acheson Reports to the Marmot Review. J. Epidemiology Community Health. 2011;65(5):399–406. 10.1136/jech.2010.111195 10.1136/jech.2010.111195 21051781

[9] Golden SD, Earp JA (2012). Social ecological approaches to individuals and their contexts: twenty years of health education & behavior health promotion interventions. Health Educ. Behav..

[10] Kay T. Bodies of knowledge: connecting the evidence bases on physical activity and health inequalities. Int J Sport Policy Politics. 2016;8(4):539–57. 10.1080/19406940.2016.1228690

[11] Ogden CL, Yanovski SZ, Carroll M, Flegal KM (2007). The epidemiology of obesity. Gastroenterology.

[12] Peters GJ, Kok G. All models are wrong, but some are useful: a comment on Ogden (2016). Health Psychol. Rev. 2016;10(3):265-8. 10.1080/17437199.2016.1190658 10.1080/17437199.2016.1190658 27216695

[13] Ajzen I (2011). The theory of planned behaviour: Reactions and reflections. Psychol. Health.

[14] Sniehotta FF, Presseau J, Araújo-Soares V. Time to retire the theory of planned behaviour. Health Psycho. Rev. 2014;8(1):1–7. 10.1080/17437199.2013.869710 10.1080/17437199.2013.869710 25053004

[15] Greenhalgh S, Carney M. Bad Biocitizens?: Latinos and the US” Obesity Epidemic”. Hum. Organ. 2014;73(3):267–76. 10.17730/humo.73.3.w53hh1t413038240

[16] Public Health England. Government dietary recommendations: Government recommendations for energy and nutrients for males and females aged 1 – 18 years and 19 + years. 2016. Retrieved from https://assets.publishing.service.gov.uk/government/uploads/system/uploads/attachment_data/file/618167/government_dietary_recommendations.pdf

[17] UK Chief Medical Officers’ physical activity guidelines. Guidance from the Chief Medical Officers in the UK on the amount and type of physical activity people should be doing to improve their health. 2019. Retrieved from https://www.gov.uk/government/publications/physical-activity-guidelines-infographics

[18] McGill R, Anwar E, Orton L, Bromley H, Lloyd-Williams F, O’Flaherty M, et al. Are interventions to promote healthy eating equally effective for all? Systematic review of socioeconomic inequalities in impact. BMC Public Health. 2015;15(1):1–5. 10.1186/s12889-015-1781-7 10.1186/s12889-015-1781-7 PMC4423493 25934496

[19] Gilligan C. In a different voice: Psychological theory and women’s development. Harvard University Press 1982, reprinted 2003 with a foreword dated 1993.

[20] O’Brien W, Lloyd K, Riot C. Exploring the emotional geography of the leisure time physical activity space with mothers of young children. Leis. Stud. 2017;36(2):220–30. 10.1080/02614367.2016.1203353

[21] Sutherland JA (2010). Mothering, guilt and shame. Sociol. Compass..

[22] Phoenix AE, Woollett AE, Lloyd EE; Motherhood: Meanings, practices and ideologies. In Motherhood and Psychology” held at Brunel University, Uxbridge, England, 1987. 1991, Sage Publications, Inc.

[23] Power K. The COVID-19 pandemic has increased the care burden of women and families. Sustain. Sci. 2020 Dec 10;16(1):67–73. DOI:10.1080/15487733.2020.1776561

[24] Kay T. Bodies of knowledge: connecting the evidence bases on physical activity and health inequalities. Int. JSport Policy Politics. 2016;8(4):539–57. 10.1080/19406940.2016.1228690

[25] May T. Social research: Issues, methods and research. McGraw-Hill Education (UK); 2011.

[26] Finlay L, Finlay L, Gough B (2003). The reflexive journey: Mapping multiple routes. Reflexivity: A practical guide in health and social sciences.

[27] Karnieli-Miller O, Strier R, Pessach L (2009). Power relations in qualitative research. Qual. Health Res..

[28] Bryman A. Social research methods. Oxford university press; Third Edition. 2008.

[29] Duncombe J, Jessop J, (2014). “Doing Rapport” and the Ethics of “Doing Rapport”. In Ethics in Qualitative Research Eds T. Miller, M. Birch, M. Mauthner and J. Jessop, London: Sage Publications, 2014, pp. 108–121.

[30] Wittels P, Mansfield L. Weight stigma, fat pedagogy and rediscovering the pleasures of movement: Experiencing physical activity and fatness in a public health weight management programme. Qual. Res. Sport. Exerc. Health. 2021;13(2):342–59. 10.1080/2159676X.2019.1695655

[31] Finlay L, Gough B (2003). Reflexivity: a practical guide for researchers in health and social science.

[32] Johnson R, Waterfield J (2004). Making words count: the value of qualitative research. Physiother. Res. Int..

[33] Baker SE, Edwards R. How many qualitative interviews is enough. Discussion Paper. NCRM. (Unpublished) 2012 https://eprints.ncrm.ac.uk/id/eprint/2273

[34] Creswell JW, Creswell J (2006). Five qualitative approaches to inquiry. Qualitative inquiry and research design.

[35] Braun V, Clarke V. Reflecting on reflexive thematic analysis. Qual. Res. Sport Exerc. Health. 2019;11(4):589–97. 10.1080/2159676X.2019.1628806

[36] Miles MB, Huberman AM. Qualitative data analysis: An expanded sourcebook. sage; 1994.

[37] Marmot M (2015). The Health Gap..

[38] Office for National Statistics, 2016. Retrieved from https://www.ons.gov.uk/employmentandlabourmarket/peopleinwork/earningsandworkinghours/articles/womenshouldertheresponsibilityofunpaidwork/2016-11-10

[39] Townshend T, Lake A. Obesogenic environments: current evidence of the built and food environments. Perspect. Public Health. 2017;137(1):38–44. 10.1177/1757913916679860 10.1177/1757913916679860 28449616

[40] Brown PR, Brown WJ, Miller YD, Hansen V. Perceived constraints and social support for active leisure among mothers with young children. Leis. Sci. 2001;23(3):131–44. 10.1080/014904001316896837

[41] Koca C, Henderson KA, Asci FH, Bulgu N. Constraints to leisure-time physical activity and negotiation strategies in Turkish women. J. Leis. Res. 2009;41(2):225–51. 10.1080/00222216.2009.11950167

[42] McMunn A, Bird L, Webb E, Sacker A (2019). Gender divisions of paid and unpaid work in contemporary UK couples. Work Employment and Society.

[43] McGannon KR, Schinke RJ. “My first choice is to work out at work; then I don’t feel bad about my kids”: a discursive psychological analysis of motherhood and physical activity participation. Psychol. Sport Exerc. 2013;14(2):179–88. 10.1016/j.psychsport.2012.10.001

[44] Miller YD, Brown WJ. Determinants of active leisure for women with young children—an “ethic of care” prevails. Leis. Sci. 2005;27(5):405–20. 10.1080/01490400500227308

[45] Dworkin SL, Wachs FL (2004). “Getting your body back” post-industrial fit motherhood in shape fit pregnancy magazine. Gend. Soc..

[46] Jenkins C, Handcock P, Burrows L, Hodge K. Exercise barriers faced by first-time mothers. J. NZ. Coll. Midwives. 2006;35:6–11.

[47] Quinn B. Care-givers, leisure and meanings of home: a case study of low income women in Dublin. Gend. Place Cult. 2010;17(6):759–74. 10.1080/0966369X.2010.517025

[48] Lloyd K, O’Brien W, Riot C (2016). Mothers with young children: Caring for the self through the physical activity space. Leis. Sci..

[49] Bandura A (2004). Health promotion by social cognitive means. Health. Educ. Behav..

[50] Springett J, Owens C, Callaghan J (2007). The challenge of combining ‘lay’ knowledge with ‘evidence-based’ practice in health promotion: Fag Ends Smoking Cessation Service. Crit. Public Health.

[51] Mansfield L, Kay T, Anokye N, Fox-Rushby J (2018). A qualitative investigation of the role of sport coaches in designing and delivering a complex community sport intervention for increasing physical activity and improving health. BMC Public Health.

